# Expression and gene regulation network of TYMS and BCL2L1 in colorectal cancer based on data mining

**DOI:** 10.7717/peerj.11368

**Published:** 2021-06-02

**Authors:** Yanghua Jie, Xiaobei Yang, Weidong Chen

**Affiliations:** 1 Department of Radiotherapy center, Affiliated Hospital of Traditional Chinese Medicine, Xinjiang Medical University, Urumqi, China; 2 Department of Anorectal, Urumqi City Hospital of Traditional Chinese Medicine, Urumqi, China; 3 Department of Anorectal, Hospital (T.C.M) Affiliated to Southwest Medical University, Luzhou, China

**Keywords:** TYMS, BCL2L1, colorectal cancer, Prognosis, Functional network analysis

## Abstract

**Background:**

The purpose of this study was to study the role of thymidylate synthetase (TYMS) and B-cell lymphoma-2 like 1 (BCL2L1) in the occurrence and development of colorectal cancer and its potential regulatory mechanism.

**Methods:**

The Cancer Genome Atlas (TCGA) and Gene Expression Omnibus (GEO) were analyzed to examine the expression and prognostic value of TYMS and BCL2L1 in colorectal cancer. C-BioPortal analysis was used to detect the TYMS and BCL2L1 alterations. Through The Human Protein Atlas (THPA), the TYMS and BCL2L1 protein levels were also assessed. The protein protein interaction (PPI) network was built using GeneMANIA analysis, while co-expression genes correlated with TYMS and BCL2L1 were identified using LinkedOmics analysis. Finally, we collected clinical samples to verify the expressions of TYMS and BCL2L1 in colorectal cancer.

**Results:**

TYMS and BCL2L1 were up-regulated, and TYMS and BCL2L1 genomic alterations were not associated with the occurrence of colorectal cancer. TYMS and BCL2L1 were significantly connected with the prognosis of colorectal cancer patients. The genes interacted with TYMS and BCL2L1 were linked to functional networks involving pathway of apoptosis, apoptosis-multiple species, colorectal cancer, platinum drug resistance and p53 signaling pathway. qRT-PCR verification results of TYMS were consistent with the result of TCGA and GEO analysis.

**Conclusions:**

This study display that data mining can efficiently provide information on expression of TYMS and BCL2L1, correlated genes of TYMS and BCL2L1, core pathways and potential functional networks in colorectal cancer, suggesting that TYMS and BCL2L1 may become new prognostic and therapeutic targets for colorectal cancer.

## Introduction

Colorectal cancer is the one of most common malignancies affecting the whole digestive tract, and is the main cause of cancer-associated mortality globally, with about 1.4 million new cases per year (Bray et al., 2018; Cojocneanu et al., 2020). Until now, the early detection or diagnosis of colorectal cancer is difficult due to the lack of effective markers and measures (Mai et al., 2018). To date, the main treatment strategies for colorectal cancer include laparoscopic surgery, radiotherapy and palliative chemotherapy (Lin et al., 2020). Despite some remarkable achievements have been achieved in recent years, most patients are diagnosed with advanced colorectal cancer, and the 5-year survival rate of patients with colorectal cancer is still unsatisfactory (Siegel, Desantis & Jemal, 2014; Siegel et al., 2017). Therefore, it is crucial to uncover the molecular mechanisms of colorectal cancer progression and to identify the novel biomarkers to improve the diagnosis, therapy and prognosis of colorectal cancer.

The study of human diseases ultimately depends on understanding the genome and its functions (Ozsolak et al., 2009). Rapid advance of high throughput technologies and bioinformatics analysis has provided novel means to study cancer in a more comprehensive fashion. Many researchers are combining omics data from different platforms to study cancer mechanisms and look for reliable biomarkers (Chen et al., 2020; Sun et al., 2020; Wang et al., 2015; Zhang et al., 2020). Accumulating numbers of reports found that some molecular markers are involved in the pathogenesis of colorectal cancer and have clinical association with colorectal cancer (Lin et al., 2020; Wang et al., 2019; Zhang et al., 2020). Based on the TCGA dataset, Lin et al., (2020) have found that 71 differentially expressed immune-related genes are associated with prognosis of colorectal cancer patients. Huang et al. (2020) have displayed that CILP2 is associated with advanced stages and could play a role as an independent predictor of poor survival in colorectal cancer. Based on the TCGA, GEO, and Oncomine databases, Liu et al., (2020) have suggested that SCARA5 expression level may be a reliable adjuvant parameter to diagnose colorectal cancer and predict tumor metastasis and prognosis. Chang et al. (2020) have reported that alterations in TMEM240 are commonly found in Western and Asian populations and can potentially be used for early prediction and as poor prognosis and early-recurrence biomarkers in colorectal cancer. With the help of the bioinformatics, we discovered novel biomarkers to elucidate the underlying mechanisms of colorectal cancer.

## Materials and Methods

### Open targets analysis

Open Targets (https://www.targetvalidation.org) is a user-friendly interface for the integration of genetics, omics, animal models, chemical data and scientific literature to score and sort target-disease associations (Carvalho-Silva et al., 2019). The database provides a target-centered workflow to identify diseases that may be associated with specific targets, or a disease-centered workflow to identify targets that may be associated with specific diseases. The platform currently integrates over 6 billion evidence from 18 public data sources and calculates nearly 3 billion associations between 21,149 human genes and 10,101 diseases and phenotypes. We utilized Open Targets to carry out the association between TYMS and BCL2L1 and gastrointestinal diseases.

### Expression of TYMS and BCL2L1 in colorectal cancer

The raw data of colorectal cancer patients were obtained from The Cancer Genome Atlas (TCGA) and Gene Expression Omnibus (GEO) datasets GSE89076. We used the TCGA and GSE89076 data to analyze expression of TYMS and BCL2L1 in colorectal cancer.

### C-BioPortal analysis

The cBio Cancer Genomics Portal (http://cbioportal.org) is an open-access web interface for interactive exploration of multidimensional cancer genomics data sets, currently providing access to data from more than 5,000 tumor samples from 20 cancer studies. The database decreases molecular profiling data from cancer tissues and cell lines to understandable genetic, epigenetic, gene expression, and proteomic events. The query interface combines customized data storage to enable researchers to explore gene changes in samples, genes and pathways interactively and to link these changes to clinical outcomes when basic data are available. We used c-BioPortal to analyze TYMS, BCL2L1 and TP53 alterations in the TCGA colorectal cancer sample. The search parameters included mutation, CNVs, and mRNA expression.

### Survival analysis

To evaluate the prognostic value of TYMS and BCL2L1 in colorectal cancer, survival analysis was built using clinical data from TCGA and GEO datasets GSE12945. Kaplan–Meier curve was plotted using the survival (https://cran.r-project.org/web/packages/survival/index.html) in R. For survival analysis, patients from TCGA dataset were divided into high−/low-risk groups according to the Cox regression model. Statistical significance was calculated using the log-rank test (*P*-values of <0.05).

### Immunohistochemistry analysis

The pictures of immunohistochemistry were obtained from the human protein atlas (THPA) (http://www.proteinatlas.org/), a publicly available database includes >5 million images of immunohistochemically stained tissues and cells (Uhlen et al., 2010). THPA version 17 is a database of tissue microarray images labeled with antibodies against 15,297 human proteins. Data regarding the expression levels of TYMS and BCL2L1 in colorectal cancer tissues were downloaded from THPA.

### GeneMANIA analysis

GeneMANIA (http://www.genemania.org) is a user-friendly web tool for building protein-protein interaction (PPI) network, generating hypotheses about gene function, analyzing gene lists and prioritizing genes for functional assays (Warde-Farley et al., 2010). The GeneMANIA was applied to build the PPI network of proteins interacted with TYMS and BCL2L1.

### Functional annotation of genes interacted with TYMS and BCL2L1

GO classification and KEGG pathway enrichment analysis of genes correlated with TYMS and BCL2L1 were performed using clusterProfiler (version 3.10.1). *P*-value < 0.05 was considered as statistically significant.

### LinkedOmics analysis

The LinkedOmics database (http://www.linkedomics.org/login.php) is a web database for analyzing cancer-associated multi-dimensional datasets of 32 cancer types and 11,158 patients (Vasaikar et al., 2018). Genes positively and negatively correlated with TYMS and BCL2L1 were obtained using LinkedOmics. The screening criteria were *P* value < 0.05 and |*r*| ≥ 0.5. Functional annotation of co-expression genes with TYMS and BCL2L1 was performed using GeneCodis3 (https://genecodis.genyo.es/). *P*-value < 0.05 was considered as statistically significant.

### qRT-PCR verification

Fourteen tissue samples from seven colorectal cancer patients and seven adjacent normal samples were obtained. This study has been approved by the ethics institute of Affiliated Hospital of traditional Chinese medicine, Xinjiang Medical University. The signed informed consents of all the participants were obtained.

Total RNA was isolated using RNA simple total RNA kit (Invitrogen, China). Fast Quant RT Kit (Invitrogen, China) was performed to obtain the complementary DNA. With Super Real PreMix Plus SYBR Green (Invitrogen, China), quantitative real-time PCR were generated using the ABI 7500 system. The 2 −ΔΔCt method was used to address the data. The human ACTB and GAPDH was used as endogenous controls for gene expression.

## Results

### The correlation between TYMS and BCL2L1 and gastrointestinal diseases

**Figure 1:**
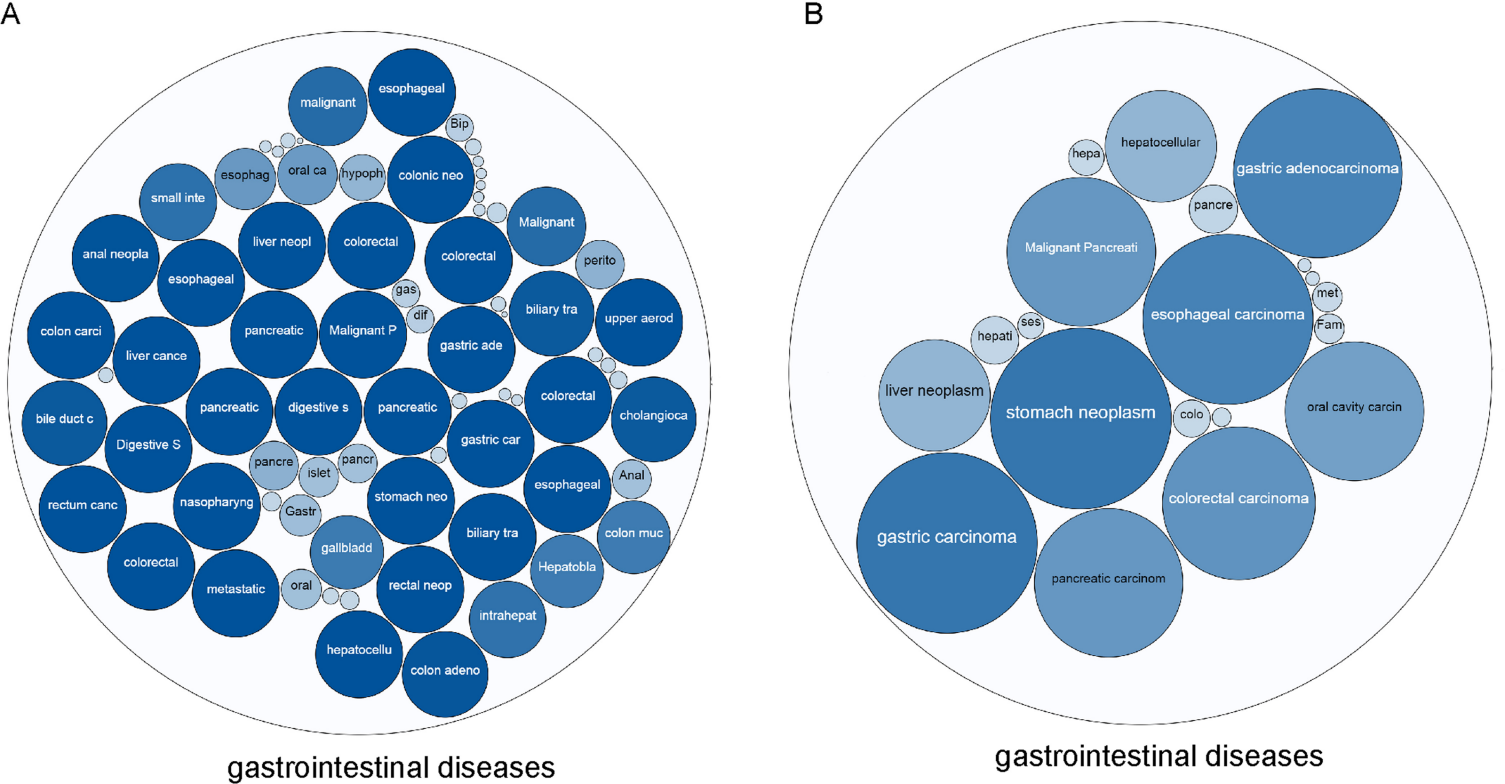
The correlation between TYMS and BCL2L1 and gastrointestinal diseases. (A) TYMS. (B) BCL2L1. The larger the bubble and the darker the color, the more associated with the disease.

Based on the Open Targets analysis, we found 76 gastrointestinal diseases related to TYMS, of which 27 diseases had a comprehensive score of 1 (score range of 0–1) (Fig. 1A). There were 23 gastrointestinal diseases related to BCL2L1, among which the top 5 were stomach neoplasm, gastric carcinoma, esophageal carcinoma, gastric adenocarcinoma, colorectal carcinoma (Fig. 1B). Among the gastrointestinal diseases targeted by both TYMS and BCL2L1, metastatic colorectal cancer ranked the first overall.

### Expression of TYMS and BCL2L1 in colorectal cancer

**Figure 2:**
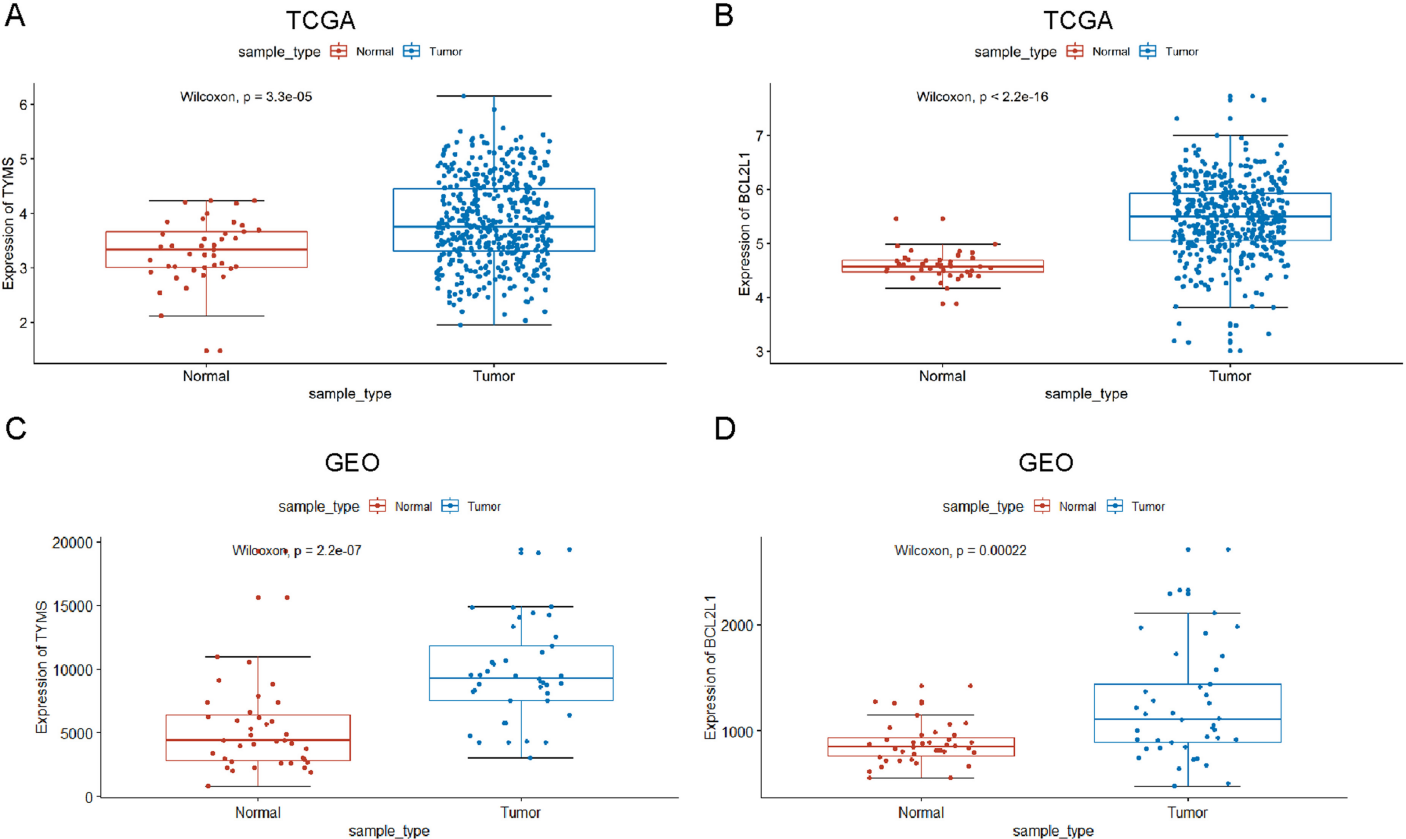
TYMS and BCL2L1 expression in patients with colorectal cancer. (A) TYMS expression level of colorectal cancer in TCGA. (B) BCL2L1 expression level of colorectal cancer in TCGA. (C) TYMS expression level of colorectal cancer in GSE89076. (D) BCL2L1 expression level of colorectal cancer in GSE89076. The *x*-axis shows sample type and *y*-axis shows a gene expression level.

Nex, we studied TYMS and BCL2L1 expression levels in multiple colorectal cancer samples from TCGA, and the results showed that TYMS and BCL2L1 were markedly up-regulated in colorectal cancer patients than normal control (Figs. 2A and 2B). We also evaluated TYMS and BCL2L1 expression in GSE89076 from GEO. The expression level of TYMS and BCL2L1 were significantly elevated in colorectal cancer patients than normal control (Figs. 2C and 2D).

### Genomic alterations of TYMS and BCL2L1 in colorectal cancer

**Figure 3:**
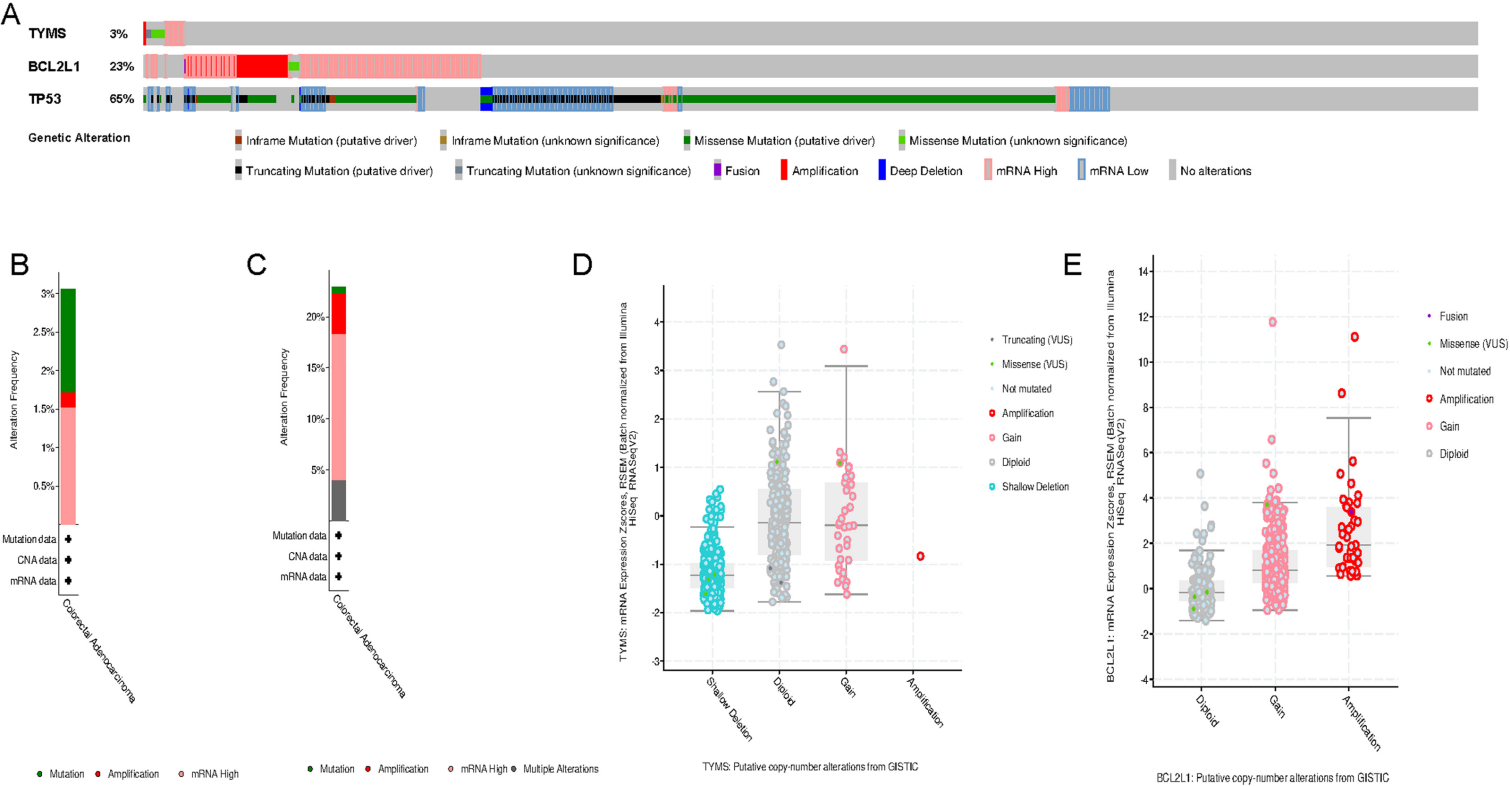
Visual summary of TYMS and BCL2L1 alterations in colorectal cancer. (A) TYMS, BCL2L1 and TP53 alterations in colorectal cancer. (B) Mutation of TYMS in colorectal cancer. (C) Mutation of BCL2L1 in colorectal cancer. (D) Putative copy-number alterations of TYMS from GISTIC. (E) Putative copy-number alterations of BCL2L1 from GISTIC.

We carried out the cBioPortal to determine the types and frequency of TYMS and BCL2L1 alterations in in colorectal cancer according to sequencing data from TCGA database. TP53 is a well-known carcinogen that causes mutations in 60% to 70% or more of solid tumors. Here, TP53 was defined as a positive control reference. TYMS was altered in 16 of 524 (3.05%) colorectal cancer patients (Fig. 3A). These alterations were mRNA up-regulation in eight cases (1.53%), amplification in one cases (0.19%) and mutation in seven case (1.34%) (Figs. 3B and 3D). BCL2L1 was altered in 120 of 524 (22.9%) colorectal cancer patients (Fig. 3A). These alterations were mRNA up-regulation in 75 cases (14.31%), amplification in 21 cases (4.01%), mutation in three case (0.57%), and multiple alterations in 21 cases (4.01%) (Figs. 3C and 3E). The results indicated that the mutation ratio of TYMS and BCL2L1 were small, suggesting that the occurrence of colorectal cancer was not associated with TYMS and BCL2L1 genomic alterations.

### Survival analysis of TYMS and BCL2L1 in colorectal cancer

**Figure 4:**
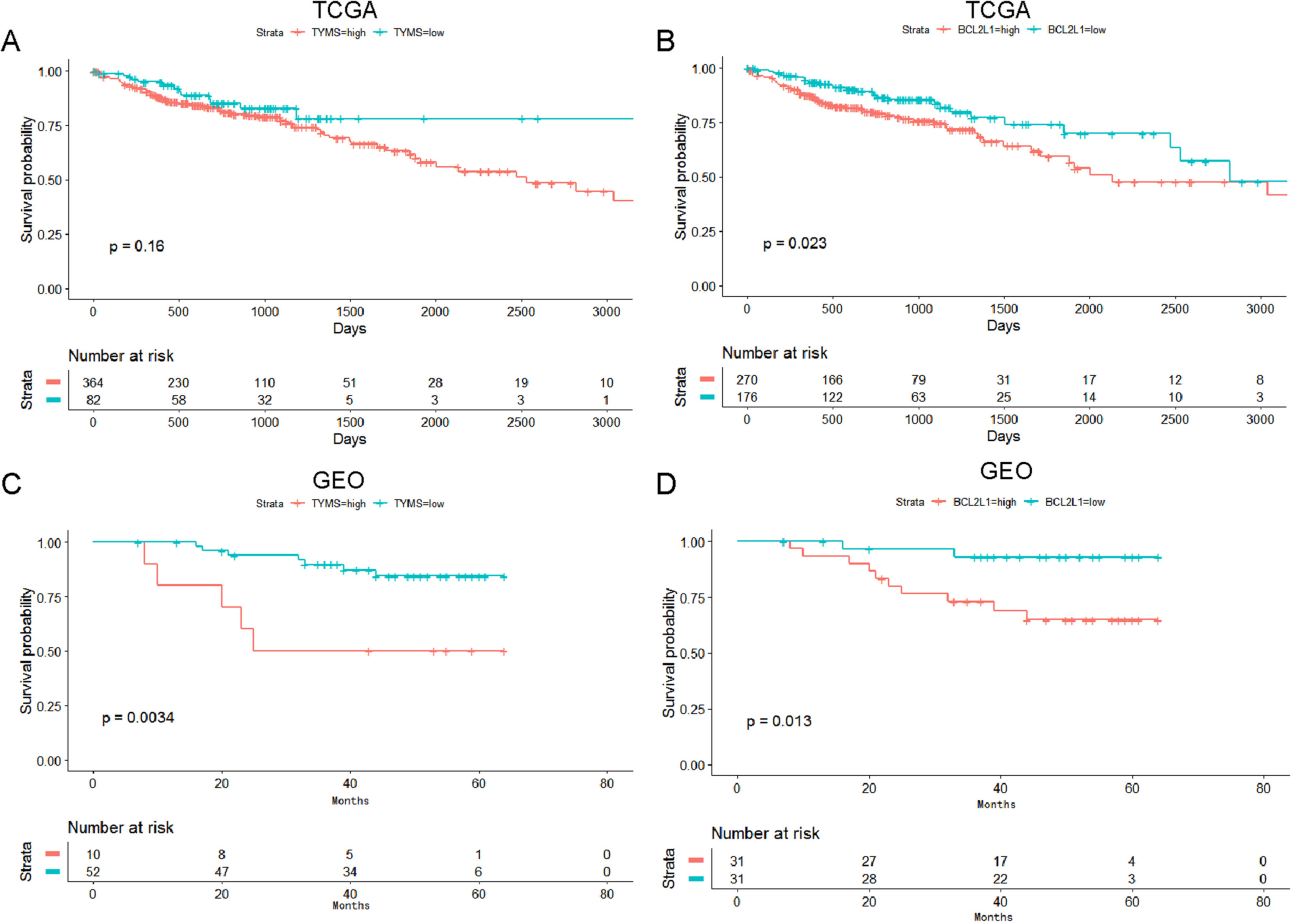
Survival analysis of TYMS and BCL2L1 in colorectal cancer. (A) The prognostic value of TYMS was analyzed using clinical data from TCGA. (B) The prognostic value of TYMS was analyzed using clinical data from TCGA. (C) The prognostic value of TYMS was analyzed using clinical data from the GSE12945 dataset. (D) The prognostic value of BCL2L1 was analyzed using clinical data from the GSE12945 dataset.

The prognostic value of TYMS and BCL2L1 was analyzed using clinical data from TCGA and GSE12945 dataset. TYMS was associated with the prognosis of colorectal cancer patients, and high expression of TYMS was markedly associated with lower survival rate in colorectal cancer patients (Figs. 4A and 4C). BCL2L1 was significantly associated with the prognosis of colorectal cancer patients, and high expression of BCL2L1 was markedly associated with lower survival rate in colorectal cancer patients (Figs. 4B and 4D).

### Immunohistochemistry analysis of TYMS and BCL2L1 in colorectal cancer

**Figure 5:**
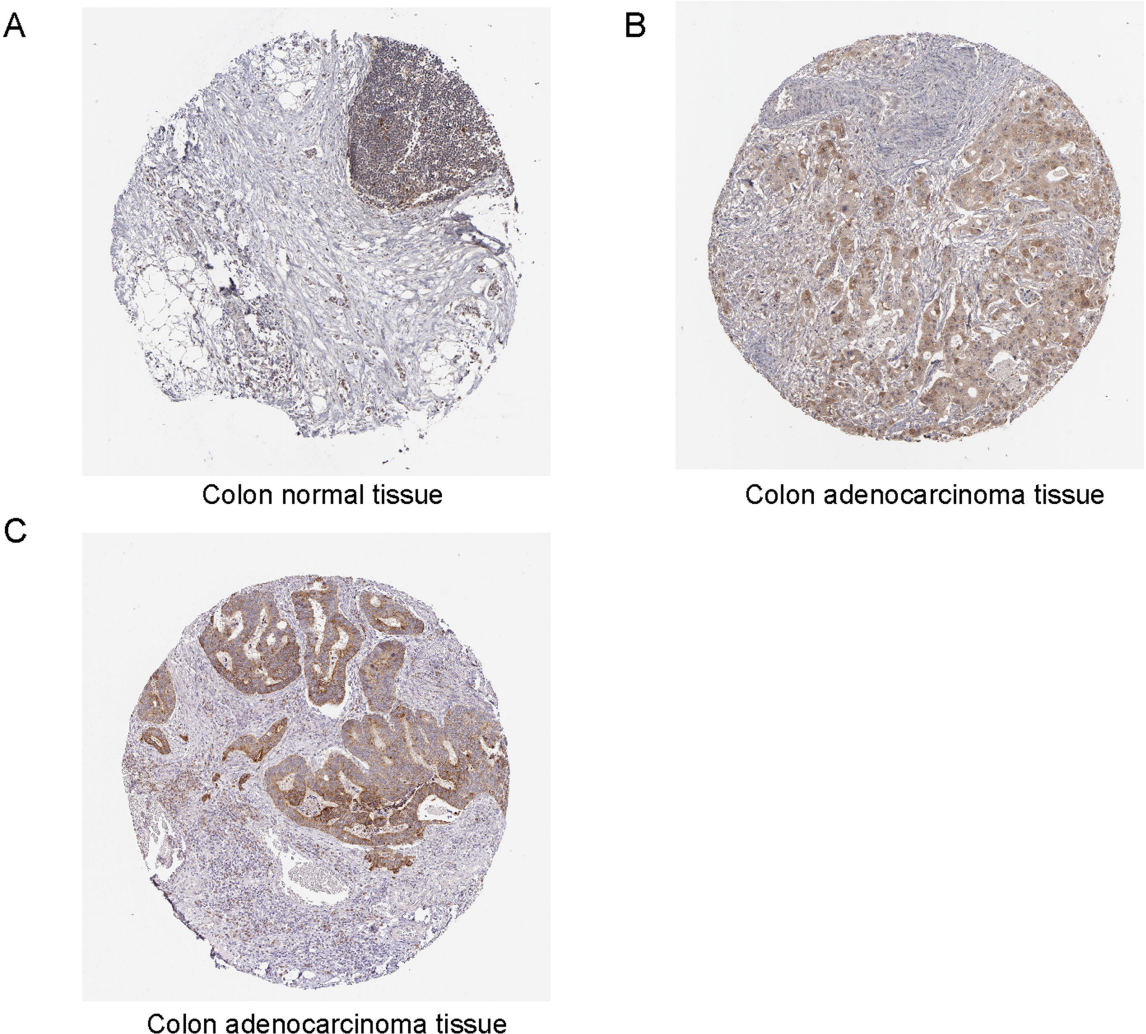
Immunohistochemistry analysis of TYMS and BCL2L1 in colorectal cancer. (A) TYMS expression level in the colon normal tissue samples. (B) TYMS expression level in the colorectal cancer specimens samples. (C) BCL2L1 in colorectal cancer specimens.

Through Human Protein Atlas (http://www.proteinatlas.org), we found the protein expression of TYMS was elevated in colorectal cancer specimens compared with colon normal tissue samples (Figs. 5A and 5B). We also downloaded the immunohistochemistry pictures of BCL2L1 in colorectal cancer specimens (Fig. 5C).

### PPI network and functional annotation of genes interacted with TYMS and BCL2L1

**Figure 6:**
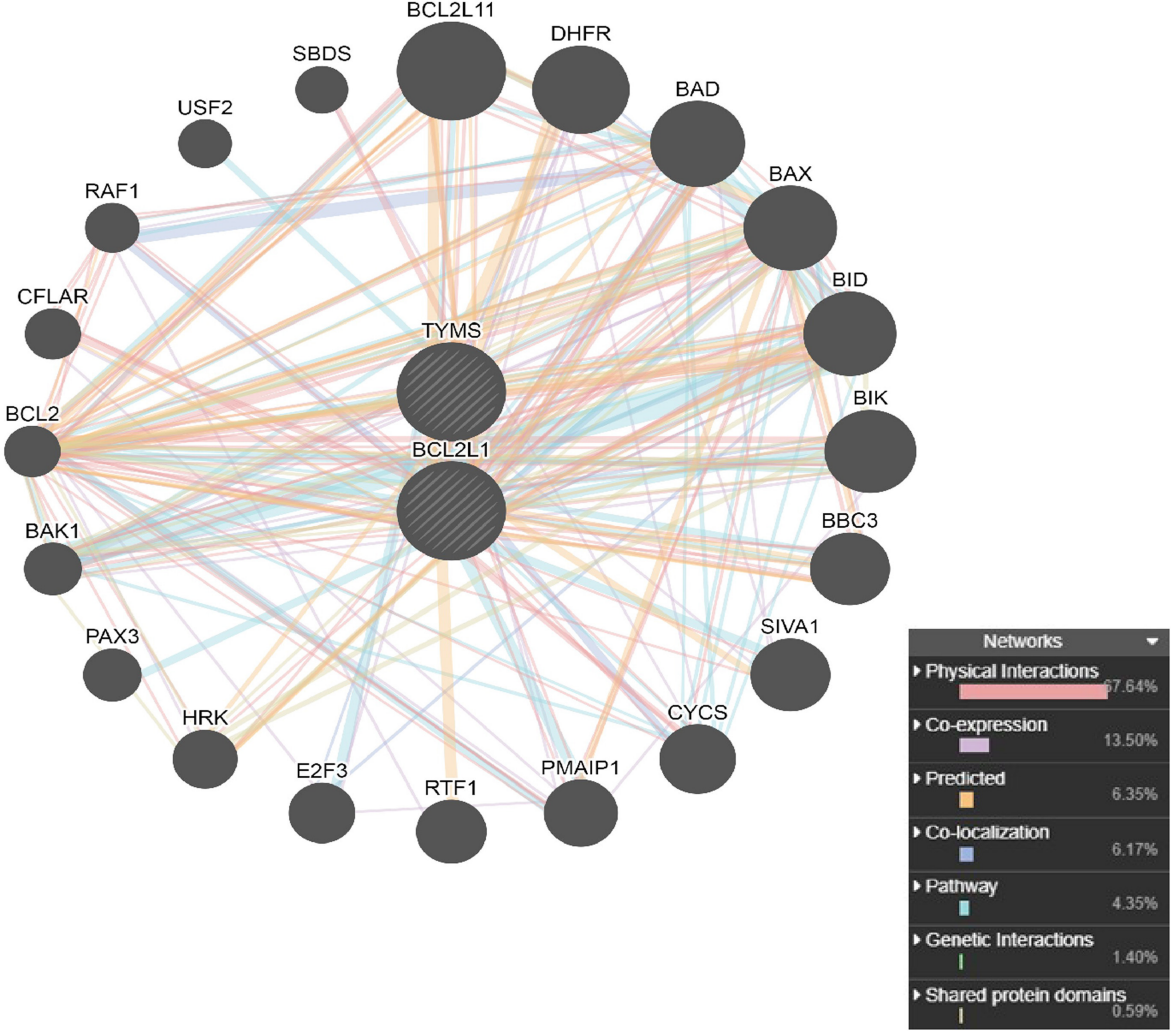
PPI network analysis of interacting genes with TYMS and BCL2L1. Circles are used to represent nodes, and lines are used to represent edges.

**Figure 7:**
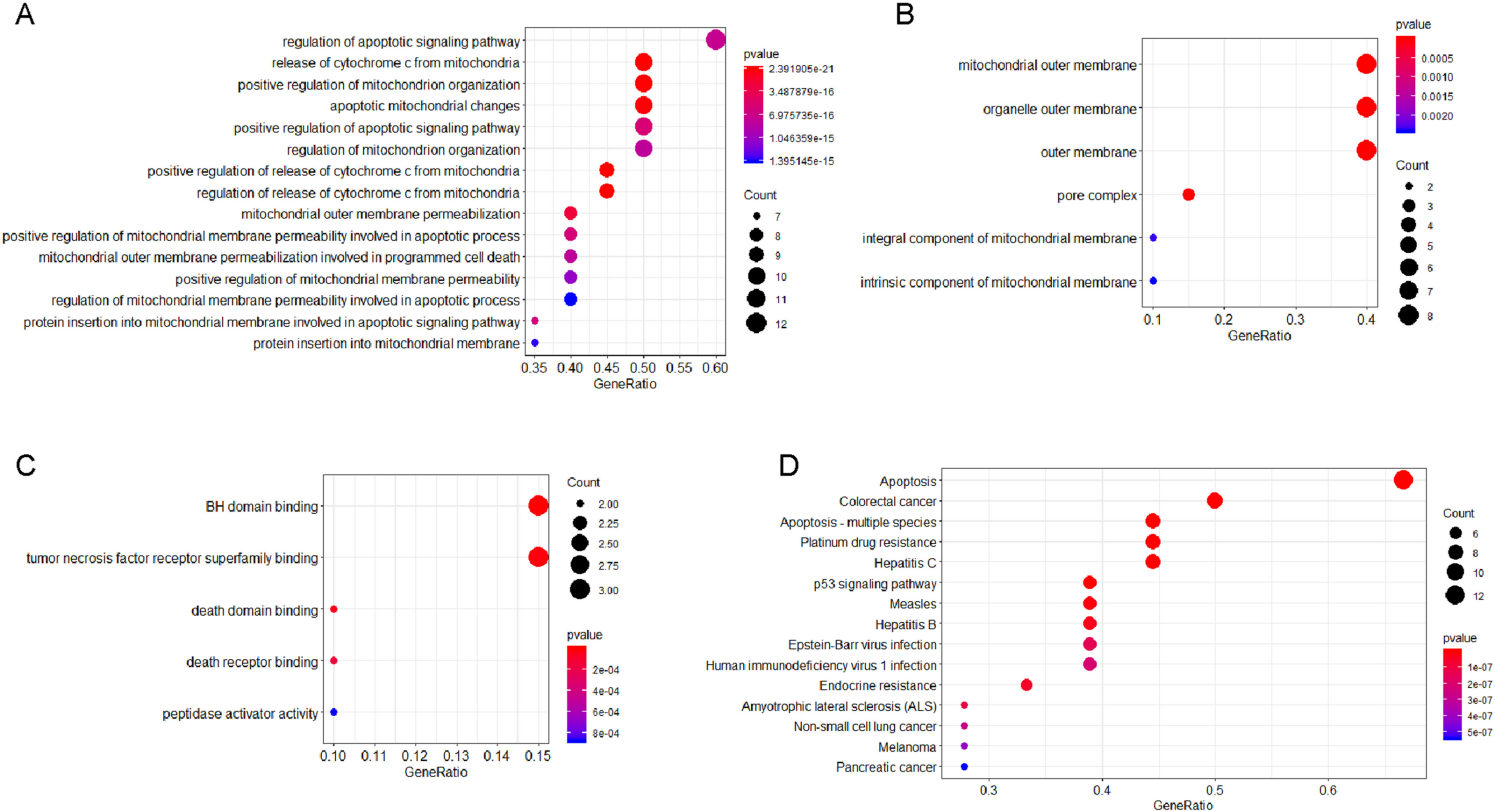
GO terms or KEGG pathways of genes interacted with TYMS and BCL2L1. The *x*-axis shows -log P, and the *y*-axis shows GO terms or KEGG pathways. (A) Biological process. (B) Cellular component. (C) Molecular function. (D) KEGG pathways.

The PPI network analysis of interacting genes with TYMS and BCL2L1 were generated by GeneMANIA. The PPI network was consisted of 22 nodes and 276 edges (Fig. 6). A total of 20 interacting genes with TYMS and BCL2L1 were applied to execute the GO and KEGG pathway enrichment analysis. GO enrichment results indicated that positive regulation of release of cytochrome c from mitochondria (*P* = 2.39E−21), release of cytochrome c from mitochondria (*P* = 7.32E−21), mitochondrial outer membrane (*P* = 4.85E−12) and BH domain binding (*P* = 1.49E−07) were markedly enriched GO terms (Figs. 7A–7C). KEGG pathway results found that apoptosis (*P* = 6.58E−18), apoptosis-multiple species (*P* = 1.14E−15), colorectal cancer (*P* = 5.96E−14), Platinum drug resistance (*P* = 1.39E−12) and p53 signaling pathway (*P* = 1.09E−10) were five significantly enriched pathways (Fig. 7D).

**Figure 8:**
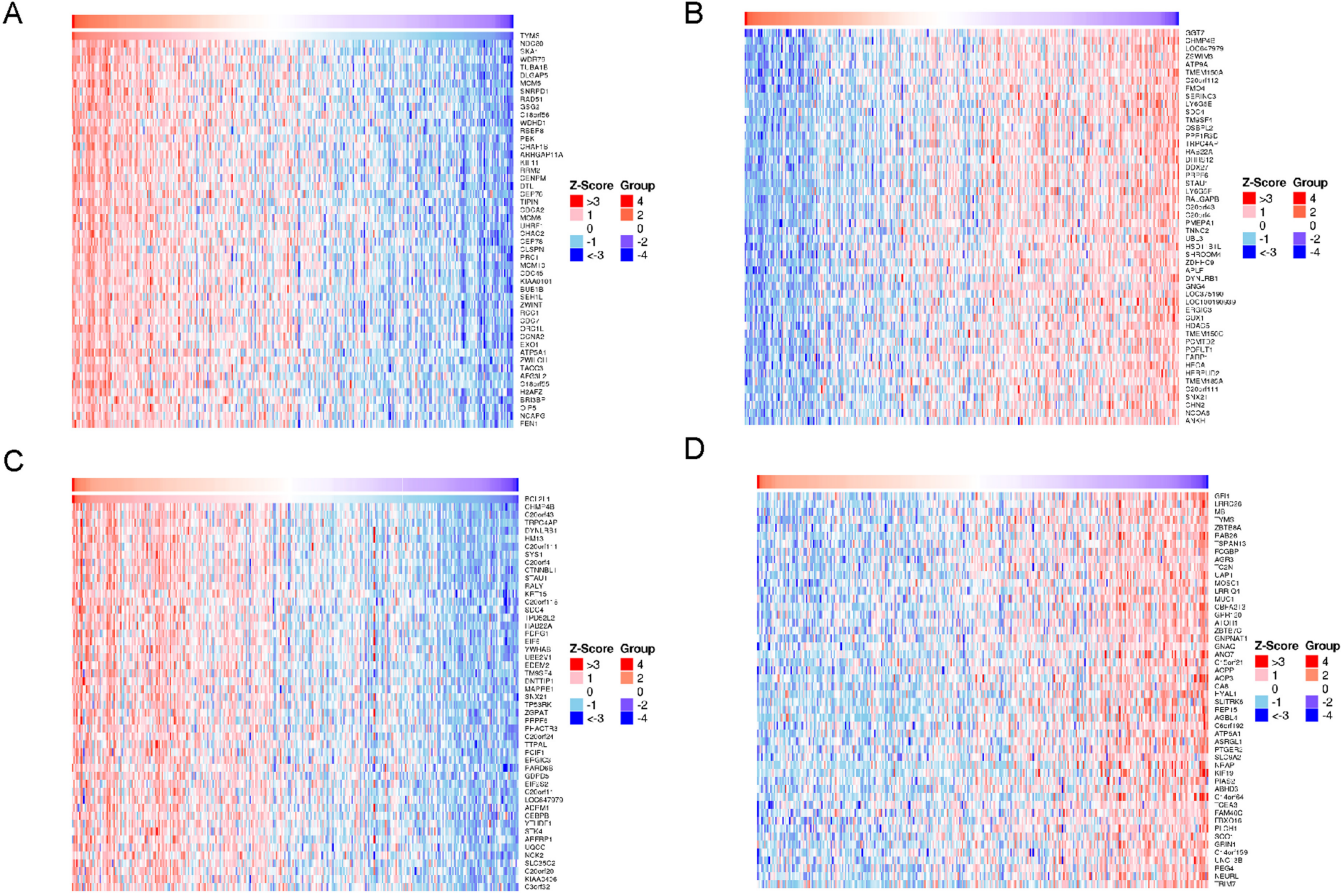
Heat maps of co-expression genes in correlation with TYMS and BCL2L1. (A) Genes positively correlated with TYMS. (B) Genes negatively correlated with TYMS. (C) Genes positively correlated with BCL2L1. (D) Genes negatively correlated with BCL2L1.

**Figure 9:**
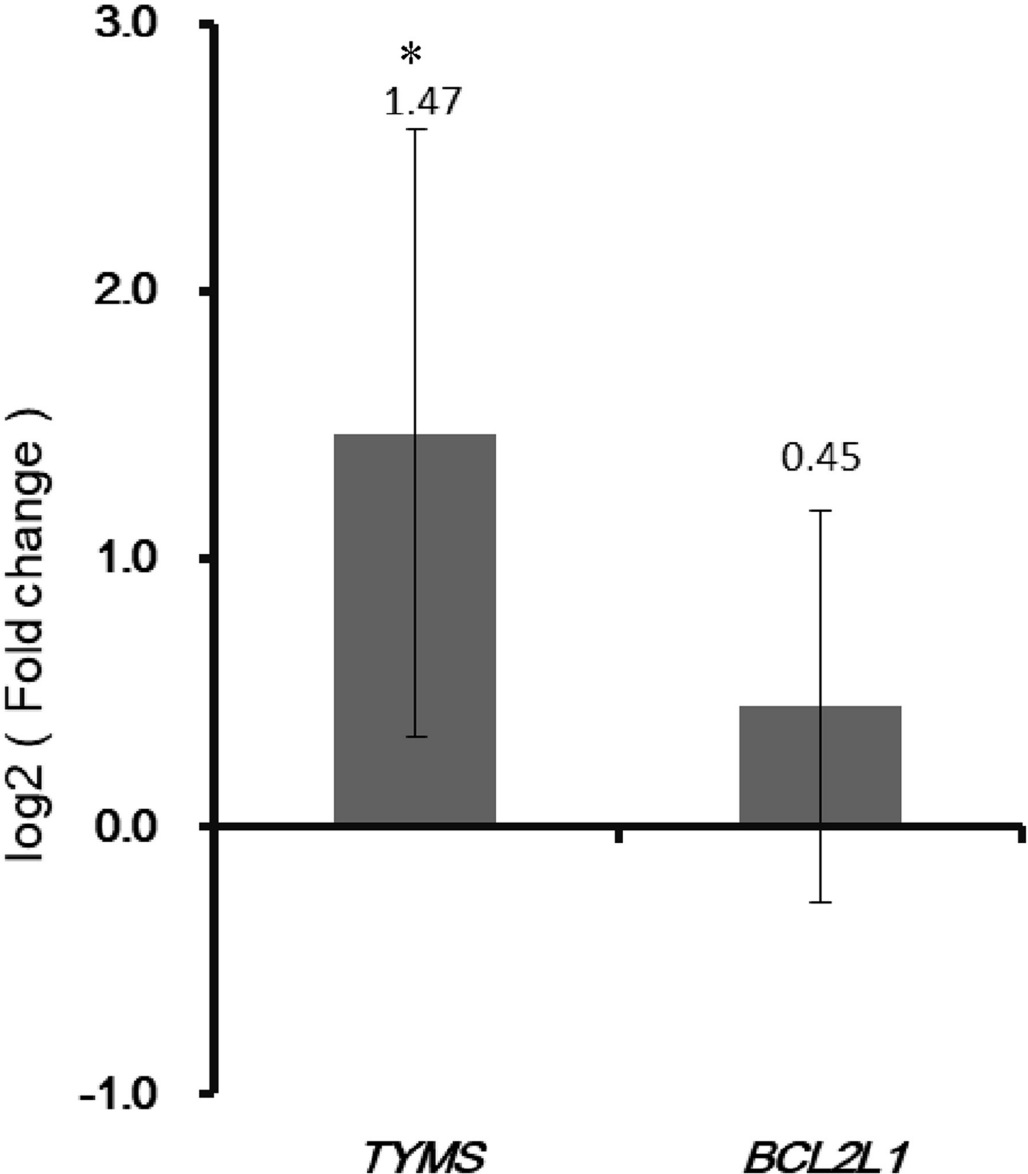
Validation the expression of TYMS and BCL2L1 by qRT-PCR. The *X*-axis represented normal adjacent and colorectal cancer groups. The *Y*-axis represented gene expression levels. * *P* < 0.05.

### Co-expression genes correlated with TYMS and BCL2L1

A total of 8,259 genes displayed significant positive correlations with TYMS, whereas 11,538 demonstrated significant negative correlations. A total of 4,340 genes displayed significant positive correlations with BCL2L1, whereas 4,457 demonstrated significant negative correlations. Hierarchical clustering analysis of 50 significant gene sets positively and negatively correlated with TYMS were shown in Figs. 8A and 8B, respectively. Hierarchical clustering analysis of 50 significant genes positively and negatively correlated with BCL2L1 were displayed in Figs. 8C and 8D, respectively.

### qRT-PCR verification

As shown in Fig. 9, the qRT-PCR verification results indicated that expression of TYMS was up-regulated in colorectal cancer tissues compared with adjacent normal tissues. However, the expression of BCL2L1 in colorectal cancer patients showed no significant change compared with adjacent normal controls.

## Discussion

Colorectal cancer is the primary cause of cancer-related death, and the treatment of colorectal cancer remains to be ameliorated. 5-fluorouracil is the main drug for clinical treatment of colorectal cancer at present, and it is the main drug for adjuvant therapy and first-line treatment of metastasis (Giuliani & Bonetti, 2016). High expression of Thymidylate synthetase (TYMS) is associated with resistance to TYMS targeted drugs such as 5-fluorouracil in colorectal cancer (Varghese et al., 2019). TYMS is located on chromosome 18p, encoding an enzyme involved in DNA replication and repair, and its function in malignant tumor cells has been reported. TYMS is found to be up-regulated in breast cancer, lung cancer, liver cancer and prostate cancer (Donner et al., 2019; Gupta et al., 2016; Russo et al., 2018; Sun et al., 2015). Recent studies have been reported that TYMS is a biomarker with diagnostic and prognostic value in a variety of cancers. High TYMS expression predicts poor prognosis after liver cancer resection, suggesting that TYMS could be a reliable predictor of prognosis in patients with liver cancer resection (Donner et al., 2019). Overexpression of TYMS in pancreatic cancer leads to a decrease in overall survival and relapse-free survival, which is a biomarker for diagnosis and prognosis of pancreatic cancer (Fu et al., 2019). In order to reveal the potential function of TYMS in colorectal cancer and its regulatory network in more detail, we conducted bioinformatics analysis of public sequencing data to guide future studies of colorectal cancer.

B-cell lymphoma-2 like 1 (BCL2L1) is a member of BCL2 family that modulates mitochondrial-induced apoptosis via suppressing the expression of proapoptotic factors (Yang et al., 2020). BCL2L1 has been reported as a key regulator in multiple cancer types including cervical cancer and colorectal cancer (Obasi et al., 2018; Yang et al., 2020; Zhang et al., 2015). High expression of let-7c leads to the activation of Bax through regulating BCL2L1, thereby increasing the release of cytochrome c and promoting the apoptosis of colorectal cancer (Zhang et al., 2018). Sillars-Hardebol et al. have reported that BCL2L1 regulates colorectal cancer progression in a chromosome 20q gain-dependent manner (Sillars-Hardebol et al., 2012). Here, we analyzed BCL2L1 expression and mutations in data from colorectal cancer patients in TCGA and GEO databases. Using multi-dimensional analysis methods, we uncovered the potential function of BCL2L1 in colorectal cancer. Herein, TYMS and BCL2L1 were up-regulated in colorectal cancer patients in TCGA and GEO databases. We collected clinical samples to verify the expressions of TYMS and BCL2L1 in colorectal cancer and results of TYMS was consistent with the result of TCGA and GEO analysis.

DNA copy number variations may have significant genomic significance, interfering with genes and altering genetic content, leading to phenotypic differences (Urrutia et al., 2018). In the current study, the mutation ratio of TYMS and BCL2L1 were small, and the development of colorectal cancer was not connected with TYMS and BCL2L1 genomic changes. We also evaluated the prognostic value of TYMS and BCL2L1 in colorectal cancer. TYMS and BCL2L1 were associated with the prognosis of colorectal cancer patients, which was consistent with previous reports of TYMS and BCL2L1 in other cancers (Fu et al., 2019; Obasi et al., 2018). Through functional annotation of genes interacted with TYMS and BCL2L1, we found that apoptosis, apoptosis-multiple species, colorectal cancer, platinum drug resistance and p53 signaling pathway were significantly enriched pathways in colorectal cancer. PMAIP1, BCL2 and BAX were genes that interacted with TYMS and BCL2L1, and they were enriched in signaling pathway of apoptosis, apoptosis-multiple species, colorectal cancer, platinum drug resistance and p53 signaling pathway. These results indicated that TYMS and BCL2L1 involved the development of colorectal cancer by regulating pathway of apoptosis, apoptosis-multiple species, colorectal cancer, platinum drug resistance and p53 signaling pathway.

## Conclusions

In TCGA and GEO, TYMS and BCL2L1 were significantly up-regulated and exhibited a moderately prognostic value for colorectal cancer.. They may be a potential as a biomarker for colorectal cancer in the future. Our study utilized the more novel bioinformatics methods to execute target gene analyses in public tumor databases. However, the sample size (seven sample per group) for qRT-PCR verification was small, which is a limitation of our study, studies of large sample size need to be performed to confirm this conclusion. This study is a pilot study and further functional experiments are needed to understand the biological functions of TYMS and BCL2L1. Therefore, in vivo and in vitro experiments were necessary to uncover the pathogenesis of TYMS and BCL2L1 of colorectal cancer in the future study.

##  Supplemental Information

10.7717/peerj.11368/supp-1 Supplemental Information 1 Raw data

10.7717/peerj.11368/supp-2 Supplemental Information 2 Co-expression genes with BCL2L1

10.7717/peerj.11368/supp-3 Supplemental Information 3 Co-expression genes with TYMS

## Additional Information and Declarations

### Data Availability

The following information was supplied regarding data availability: The datasets analyzed for this work are available from public databases: The Cancer Genome Atlas (TCGA) from UCSC Xena (GDC TCGA Colon Cancer (COAD)), The Open Targets Platform (20 diseases associated with BCL2L1, 97 diseases associated with TYMS), The cBio Cancer Genomics Portal (Colorectal Adenocarcinoma (TCGA, PanCancer Atlas)), The Human Protein Atlas (THPA) (ENSG00000176890-TYMS; ENSG00000171552-BCL2L1), GeneMANIA (https://genemania.org/search/homo-sapiens/TYMS/BCL2L1/), the LinkedOmics database and the Gene Expression Omnibus (GEO) (GSE12945 and GSE89076). - GDC TCGA Colon Cancer (COAD)
https://xenabrowser.net/datapages/?dataset=TCGA-COAD.htseq_counts.tsv&host=https
- 20 diseases associated with BCL2L1
https://www.targetvalidation.org/target/ENSG00000176890/associations?fcts=therapeutic_area:EFO_0010282&view=t:bubbles
- 97 diseases associated with TYMS
https://www.targetvalidation.org/target/ENSG00000171552/associations?fcts=therapeutic_area:EFO_0010282&view=t:bubbles
- Colorectal Adenocarcinoma (TCGA, PanCancer Atlas)
http://www.cbioportal.org/results/oncoprint?genetic_profile_ids_PROFILE_MUTATION_EXTENDED=coadread_tcga_pan_can_atlas_2018_mutations&genetic_profile_ids_PROFILE_COPY_NUMBER_ALTERATION=coadread_tcga_pan_can_atlas_2018_gistic&genetic_profile_ids_PROFILE_MRNA_EXPRESSION=coadread_tcga_pan_can_atlas_2018_rna_seq_v2_mrna_median_Zscores&cancer_study_list=coadread_tcga_pan_can_atlas_2018&Z_SCORE_THRESHOLD=2.0&RPPA_SCORE_THRESHOLD=2.0&data_priority=0&profileFilter=0&case_set_id=coadread_tcga_pan_can_atlas_2018_3way_complete&gene_list=TYMS
- ENSG00000176890-TYMS
https://www.proteinatlas.org/ENSG00000176890-TYMS/pathology/colorectal+cancer#img
- ENSG00000171552-BCL2L1
https://www.proteinatlas.org/ENSG00000171552-BCL2L1/pathology/colorectal+cancer#img
- TYMS/BCL2L1
https://genemania.org/search/homo-sapiens/TYMS/BCL2L1/
- LinkedOmics database. Data from this database can be found in the Supplemental Information
http://www.linkedomics.org/login.php
